# Microbial Dysbiosis in Colorectal Cancer (CRC) Patients

**DOI:** 10.1371/journal.pone.0016393

**Published:** 2011-01-27

**Authors:** Iradj Sobhani, Julien Tap, Françoise Roudot-Thoraval, Jean P. Roperch, Sophie Letulle, Philippe Langella, Gérard Corthier, Jeanne Tran Van Nhieu, Jean P. Furet

**Affiliations:** 1 Department of Gastroenterology and LIC-EA4393, APHP and UPEC Université Paris-Est Créteil, Créteil, France; 2 INRA, UMR1319, Micalis Institute, Jouy-en-Josas, France; 3 Santé Publique - Statistique, APHP and Université Paris 12, Créteil, France; 4 CIC 006 et Plateforme de Ressources Biologiques, INSERM, Groupe Hospitalier Henri Mondor, Créteil, France; 5 Department of Pathology, APHP and Université Paris 12, Créteil, France; Lille 2 University, France

## Abstract

The composition of the human intestinal microbiota is linked to health status. The aim was to analyze the microbiota of normal and colon cancer patients in order to establish cancer-related dysbiosis.

**Patients and Methods:**

Stool bacterial DNA was extracted prior to colonoscopy from 179 patients: 60 with colorectal cancer, and 119 with normal colonoscopy. Bacterial genes obtained by pyrosequencing of 12 stool samples (6 Normal and 6 Cancer) were subjected to a validated Principal Component Analysis (PCA) test. The dominant and subdominant bacterial population (*C. leptum*, *C. coccoides*, *Bacteroides/Prevotella*, *Lactobacillus/Leuconostoc/Pediococcus groups*, *Bifidobacterium genus*, and *E. coli,* and *Faecalibacterium prausnitzii* species) were quantified in all individuals using qPCR and specific IL17 producer cells in the intestinal mucosa were characterized using immunohistochemistry.

**Findings:**

Pyrosequencing (Minimal sequence 200 nucleotide reads) revealed 80% of all sequences could be assigned to a total of 819 taxa based on default parameter of Classifier software. The phylogenetic core in Cancer individuals was different from that in Normal individuals according to the PCA analysis, with trends towards differences in the dominant and subdominant families of bacteria. Consequently, All-bacteria [log_10_ (bacteria/g of stool)] in Normal, and Cancer individuals were similar [11.88±0.35, and 11.80±0.56, respectively, (P = 0.16)], according to qPCR values whereas among all dominant and subdominant species only those of *Bacteroides/Prevotella* were higher (All bacteria-specific bacterium; P = 0.009) in Cancer (-1.04±0.55) than in Normal (-1.40±0.83) individuals. IL17 immunoreactive cells were significantly expressed more in the normal mucosa of cancer patients than in those with normal colonoscopy.

**Conclusion:**

This is the first large series to demonstrate a composition change in the microbiota of colon cancer patients with possible impact on mucosal immune response. These data open new filed for mass screening and pathophysiology investigations.

## Introduction

The human colon contains up to 10^14^ bacteria [1]. They play a major role in the fermentation of residual food, the modulation of gut immune function, and protection against pathogens and diseases [2]–[5]. Although the intestinal microbiota is largely beneficial, changes in bacterial populations or in the products of bacterial metabolism may contribute to disease.

In 1971, a study intended to identify associations between human microbiota composition and colorectal carcinogenesis, but it had to be abandoned because of technical difficulties. Later, Moore and co-workers reported that 13 bacterial species were significantly associated with a high risk of colon cancer and the Western diet [1]. However, their results were somewhat unconvincing because they investigated a small number of subjects and no intestinal investigation i.e. radiology or colonoscopy was performed. Nonetheless, since this study was carried out, the human colonic microbiota has emerged as a major environmental factor that appears to modulate the risk of colonic cancer, and dysbiosis in the gut microbiota is now believed to be a factor underlying the development of disease in genetically-predisposed individuals. However, there is no evidence whether dysbiosis does indeed occur in colon cancer.

Only a restricted set of bacterial populations in the nature have been identified in the human body and about 80% of the human bacteria identified by molecular tools i.e. metagenomic sequencing, are considered uncultivable [6]. Although some prevalent bacterial species in normal individuals are now identified by using whole genome sequencing [7], more than 60% of species remain unknown and there is no data on how dysbiosis, if any, may occur in colon cancer patients. Thus, DNA sequencing that targets hypervariable regions within small ribosomal-subunit RNA genes, especially 16S rRNA genes has made it possible to characterize the biodiversity of the microbiota, which could lead to diseases (for a review, see ref [8]). The 16S rRNA gene is a ribosomal component that is conserved in all bacteria, and it contains variable sequences that confer species specificity. According to this technique predominant taxa in the human intestinal microbiota are reported to be *Clostridium leptum,* C*lostridium coccoides,* the *Bacteroides/Prevotella* groups and the *Bifidobacterium* genus [9]. The real-time quantitative PCR (qPCR) approach has been adapted to evaluate these bacterial populations in large numbers of samples [10]–[11], and changes in microbiota components can now be studied in relation to health/disease status. Species involved will impact experimental and metabolic studies with new pathophysiology approaches. For example, *Bacteroides* populations and more specifically those of *Bacteroides fragilis*, have recently been shown to produce a metalloprotease in colon cancer patients, but not in controls [12]. This bacteria species has been shown to induce mucosal regulatory T-cell responses in the intestine involving TH17 cell recruitment in animals [13]–[14] suggesting strongly they may alter homeostasis of effector helper T-cell populations in the gut [14]–[15].

By using pyrosequencing technique, we report evidence that colon cancer disease is linked with dysbiosis mainly due to a change in dominant and subdominant species. By using qPCR, we compared intestinal bacterial communities in normal individuals and in those with colon cancer in the largest series so far reported and show level of dysbiosis on dominant microbiota species. We put these results in perspective with mucosal immune homeostasis and stool marker for mass screening.

## Results

### Characteristics of individuals

They were classified as follows: Normal (n = 119), who had normal colonoscopy; those with colon (n = 44) or rectal (n = 16) cancer (total n = 60). Patients with cancer were gender-matched (obviously two normal for one cancer) but were 10 years older than those with normal colonoscopy (Table 1) like in our cohort (Table S1 in Supplementary File S1) in because consecutive individuals were included. Normal individuals less often reported a previous personal history of polyps or a history of colon cancer in their family and did not display difference concerning BMI. Thus, apart from this item and age, they were matched for main other characteristics including BMI and food or medicine uptake with the cancer patient group (Table 1, Table S1 and Figure S1 in Supplementary File S1).

**Table 1:** Characteristics of the individuals included in the current study N = 179.

	Normal	Cancer^1^	p (lin)
***Colonoscopy + Pathology***	N = 119	N = 60	
Age: (mean ± SD)	55.8±11.6	67.1±11.6	0.001
Gender, M: n (%)BMI: (mean± SD)	55 (46.2)25.1 (0.47)	31 (51.6)24.5 (0.85)	0.140.68
Past history of polyps: n (%)	27 (22.7)	4 (6.6)	0.001
***Colon cancer in relatives***			
Yes, n (%)	67 (0.5)	16 (9.0)	0.003
Diabetes, yes n (%)	12 (10.1)	9 (15)	0.13
Hypercholesterolemia, n (%)	31 (26.4)	21 (35)	0.09
***Particular nutriment*** 2			
Regimen Yes, n (%)	21 (17.6)	11 (18.3)	0.26
Treatment, any^3^, n (%)	93 (78.1)	45 (75)	0.26
***Reason for colonoscopy: n (%)***			
Screening	44 (36.9)	10 (16.6)	
Control for polyps	22 (18.5)	4 (6.6)	<0.001
Symptoms	53 (44.6)	46 (76.6)	

1 - includes invasive cancers (n = 53), advanced adenomas (n = 2) and large villous tumours of at least 3 cm in diameter (n = 5);

2 - includes those individuals who are under any particular regimen (diabetes, vegetarian, hyper proteic, hyper vitaminic etc…);

3 - No antibiotics.

### Phylogeny issue based on taxon distribution

**Figure 1:**
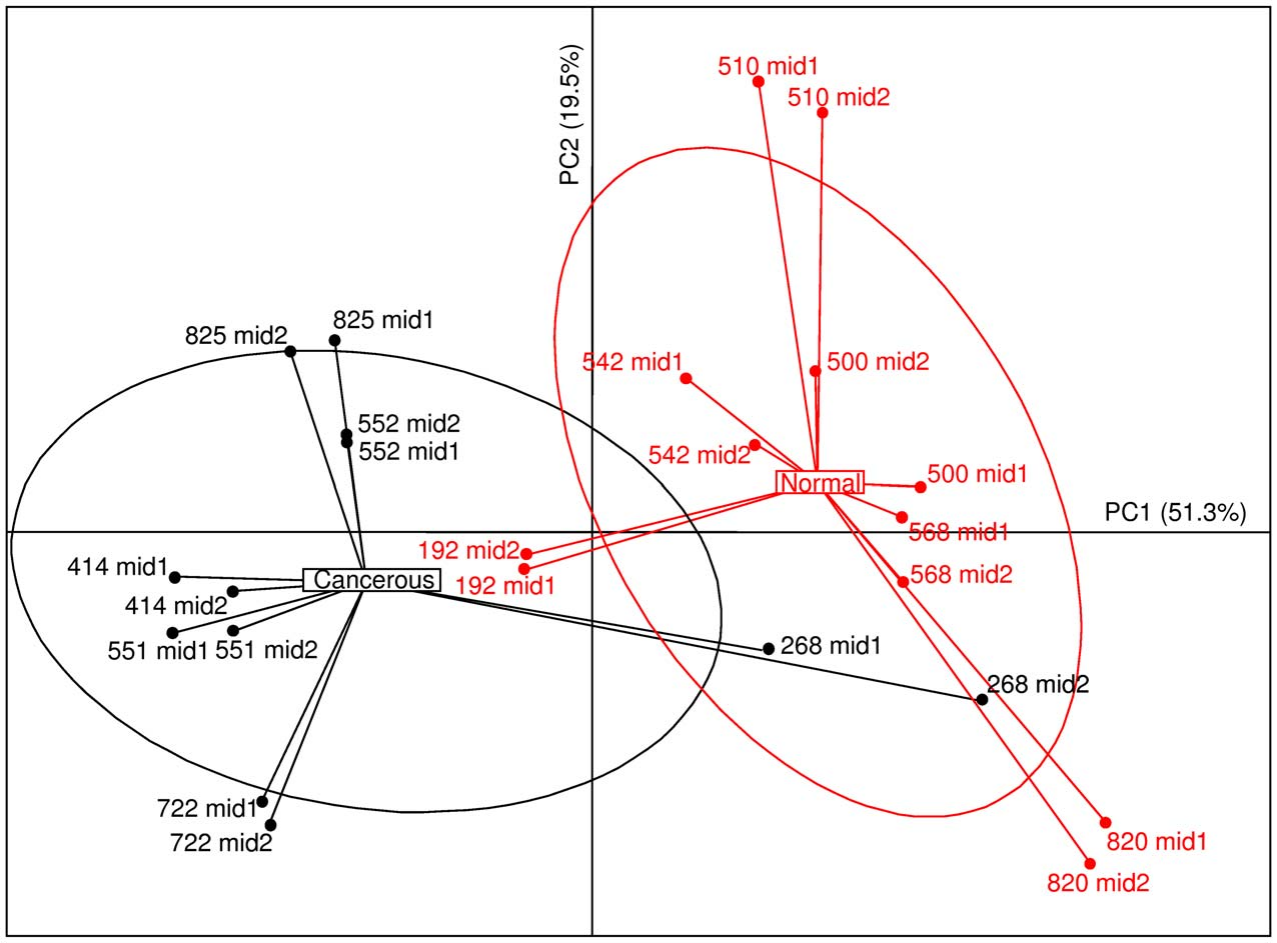
Bacterial genera abundance differentiates cancer patients and normal colonoscopy individuals. Principal component analysis, based on the 16S rRNA gene sequence abundance of 7 discriminates genera which represented at least 1% of microbiota abundance, was carried out with 6 healthy individuals (N) and 6 cancer* patients (Ca) with two replicates (noted as mid1 and mid2). Two first components (PC1 and PC2) were plotted and represented 70.83% of whole inertia. Individuals (represented by their sample id) were clustered and centre of gravity computed for each class. * They all have been selected from stage I-II of TNM classification (see also Tables S2 and S3 and Figures S2&S3 in the supplementary File S1).

From all dataset, 1,210,781 trimmed sequences were obtained and 978,710 of these could be assigned to a total of 819 taxa (Table S2 in the Supplementary File S1). A rarefaction of data was performed and gave adequate representation of the diversity of the gut microbial community (Tables S1, Table S2 and Figure S3 in the Supplementary File S1). We identified 18 bacterial genera with an abundance of more than 1%, and these genera included 75% of the sequences. Thirteen out of the 18 genera (*Alistipes, Collinsella, Bacteroides, Lachnospira, Prevotella, Subdoligranulum, Dorea, Faecalibacterium, Roseburia, Coprococcus, Streptococcus, Bifidobacterium* and *Ruminococcus)* corresponded to the human intestinal microbiota phylogenetic core [16]. PCAIV analysis also showed that about 5% of the variability could be attributed to the disease status of each sample (Normal versus Cancer; p<0.05), and the taxa indicative of the microbiota of normal and cancer individuals were clearly distinguished (Figure 1 and Figure S2 in supplementary File S1). Furthermore, 55 out of the 66 bacterial species belonging to the previously-described phylogenetic core were detected in these samples. The Monte Carlo test showed that more than 7% of the variability was impacted by health status (Normal versus Cancer; p<0.05). The variation within person was low and negligible compared to between person-variation (Figure 1 and Table S4 in supplementary File S1).

### Comparison of bacterial populations in the stools

All-bacteria levels did not differ in the Normal and Cancer groups (Table 2), whereas a significant difference was observed for *Bacteroides/Prevotella* group. This difference was related to the elevated level of these bacteria in Cancer as compared to Normal groups (Table 2). Taking all individuals together *Bacteroides/Prevotella* group density level was not linked to the age or BMI (r = 0.05; p = 0.46; see also Figure S1 in supplementary File S1). Taking all individuals with cancer the bacterial levels were not linked with tumor size or tumour staging referring to the international TNM classification (Figure S1 in supplementary File S1) and small invasive carcinomas could be associated with high levels of bacterial density. The levels of *Bacteroides/Prevotella* group were not influenced by other patient characteristics, such as age, BMI, the reason for the colonoscopy, the previous history of polyps or of cancer in their family (Table S1 in supplementary File S1), with size or location (left- versus right sided, or rectal versus colon) of the cancer (Table 2). For the other dominant or subdominant bacteria i.e. *C. leptum* group, *C. coccoides* group, the *Lactobacillus/Leuconostoc/Pediococcus* groups, the *Bifidobacterium* genus, *E. coli,* and *Faecalibacterium prausnitzii* species, we did not find any difference between patients versus Normal colonoscopy individuals.

**Table 2:** Composition of microbiota regarding dominant and sub dominant bacteria groups according to the colonoscopy and pathology results.

	Control	Cancer	*p-*value
N	119	60	-
All -bacteria*	11.88±0.35	11.80±0.56	0.21
*Clostridium/Leptum*group†	−0.002±0.024	+0.004±0.016	0.27
*Clostridium/Coccoides*group†	−1.23±0.45	−1.29±0.41	0.36
*Bacteroides/Prevotella*group†	−1.40±0.83	−1.04±0.55	0.009
*E. coli*species†	−3.74±1.28	−3.66±1.34	0.25
*Bifidobacterium*genus†	−2.03±1.22	−1.91±1.06	0.90
*Lactobacillus/Leuconostoc/* *Pediococcus*group†	−2.32±0.95	−2.24±0.85	0.27
*Faecalibacterium/prausnitzii*species† ‡	−1.05±1.02	−0.84±0.80	0.72

n: represents the numbers of studied samples.

*All-bacteria results obtained by qPCR were expressed as mean of the log10 value ± SD.

† Results were expressed as mean of normalized values ± SD,

calculated as the log number of targeted bacteria minus the log number of all-bacteria.

‡ *Faecalibacterium prausnitzii* is the major component of the *Clostridium leptum* group.

### IL17 immune cells and *Bacteroides* in the mucosa

**Figure 2:**
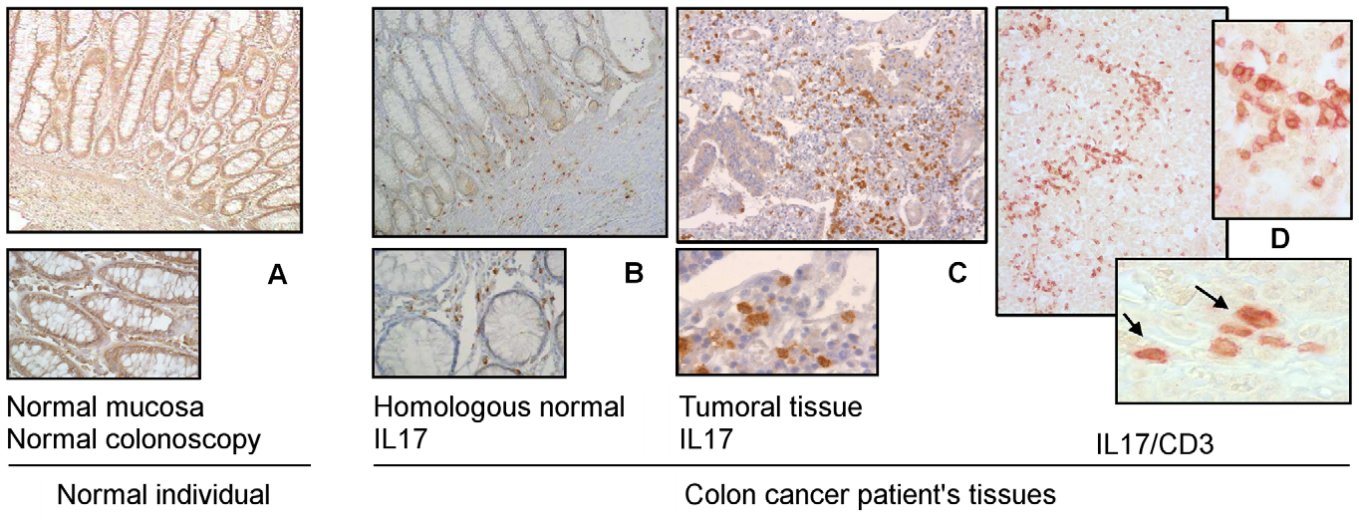
Tissue samples are immunostained by using specific antihuman IL17 goat antibody (1∶40 concentration at room temperature for 1 h) and revealed by DAB system and counterstained with haematoxylin with high magnifications in the windows. Samples from the same individuals and colonic sites were submitted to DNA extraction and PCR. Interleukin 17 (IL17)-immunoreactive cells in colonic tissues were mainly located in the *lamina propria* in the normal tissues [A: colonic normal mucosa from a normal individual (high magnification x40 at the bottom), B: colonic normal mucosa from a patient with colon cancer (high magnification x40 at the bottom)] and infiltrated the tumour tissue in a the same individual than in B [C: IL17 imlmunoreactive cells infiltrating the tumour with high magnification x40 at the bottom & D: In this double staining IL17 and CD3, the goat anti-human IL-17 antibody was added first before staining with Naphthol/Fast (red) followed by the rabbit anti-human CD3 antibody that was revealed with DAB substrate (brown); this showed that CD3 was not the only cell producing IL-17.

**Figure 3:**
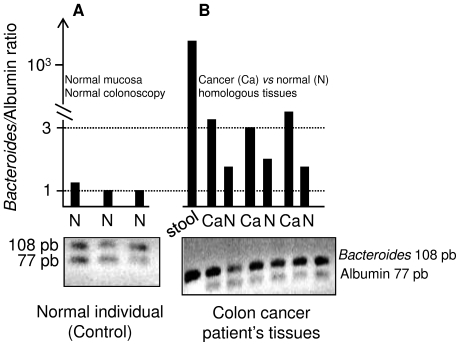
The PCR products corresponding to *Bacteroides* (108 pb) appeared to be similar to those of human Albumin (77 pb) in the tissue but highly elevated in stool samples. In the normal individual's tissue (A) gel migration system shows *Bacteroides*/Albumin ratios close to 1 whereas they appeared higher in homologous normal (N) or tumour (Ca) mucosa in the colon cancer patient's tissues (B). Note that *Bacteroides* gene amplification product in stool is dramatically higher than that detected from mucosal DNA; amplification is referred to the human Albumin gene.

These cells were found infiltrating majority of tumour samples (score ++ to +++) and in the *lamina propria* of homologous normal mucosa (score + to ++) in cancer patients' tissue samples while they were rarely or not detected in the normal mucosa (0 to +/-) in normal individuals (Figure 2). No parametric statistical test showed significant higher IL17 immunostained cell score in normal mucosa of colon Cancer patients than in Normal colonoscopy individuals (median +/- versus ++; p<0.5). After double (CD3 and IL17) staining of serial tissue sections, the majority of IL17 immunoreactive cells were found to be of CD3 marker in both (Normal mucosa or homologous to cancer normal mucosa) cases although all CD3 cells were not immunostained with IL17 antibody. However, IL17/CD3 ratios in normal colon mucosa appeared no significantly different between Normal and Cancer colonoscopy individuals (Figure 2C, D). These semi quantitative immune cell findings were linked with specific tissue adherent *Bacteroides* density (Figure 3). *Bacteroides* gene amplification product was found 1000-fold more expressed in the stool than in colon tissue samples as revealed by qPCR (Figure 3B). Furthermore, the *Bacteroides* amplification product was significantly higher in colon cancer patients' tissues (normal as well as tumoral) than in normal individuals' tissues as assessed by qPCR and revealed by gel analysis (Figure 3 and Figure S4 in supplementary File S1). In colorectal cancer patients, *Bacteroides* gene amplification product was significantly higher in tumour tissue than in normal homologous tissue (Figure S5 in supplementary File S1).

## Discussion

We report differences in the colon microbiota in individuals with colon cancer versus those with a normal colonoscopy. We showed that the distribution of bacterial genera in the microbiota varied, depending on their disease status, and qPCR revealed significant elevation of the *Bacteroides/Prevotella* population in cancer patients that appeared to be linked with elevated IL17 producing cells in the mucosa of individuals with cancer.

This study compares individuals presenting with a normal or diseased colonoscopy. Although those with a normal colonoscopy were not healthy volunteers, they can be considered to constitute a meaningful control group. This is because they were randomly selected from amongst consecutive individuals who had been referred for colonoscopy that was found to be normal. In order to reduce the bias we selected 2 normal individuals for 1 cancer case. Their characteristics matched those of the patients in the cancer group, except for their age and cases of polyps or cancer in relatives. Age differences reflect the epidemiological data in the literature. Bacterial dysbiosis found in our study was clearly independent from age. None of microbial differences observed in this study was linked with age. Another difference between cases and controls concerns higher prevalence of neoplasms in colon cancer patients' relatives. This might reflect the role of environmental factors rather than germinal genetic alterations since none of patients had stigmata of Lynch or polyposis adenomatous familial PAF syndrome diseases.

High throughput sequencing techniques for human microbiota has substantially contributed to revealing a difference in the bacterial phylogenetic core in normal individuals and those suffering from various diseases [7]. However this approach did not include cancer disease. In order to verify whether phylogenetic core was different in healthy individuals and cancer patients, 16S rRNA genes have been targeted by pyrosequencing as an alternative to the all bacteria genome sequencing. Indeed, the V3-V4 variable region of 16S rRNA can be used to provide a bacterial classification of the human microbiota [17] on the basis of pyrosequencing. By using this technology, we clearly identified more than 40,000 informative sequences on V3-V4 16S rRNA gene region from each stool sample, in the present study and this led to the construction of the phylogenetic core of microbiota [16]. This phylogenetic core was found to be different in cancer patients versus normal individuals. The differences concerned particularly dominant and sub dominant bacterial populations. It is very unlikely that the differences we found by pyrosequencing could be epiphenomenal, since all the patients were included through a standardized procedure (gender and age-matched, conditions of stool sampling and DNA extraction) and sequence similarities between duplicates (within person-variation) was very high. Consequently, main groups, genus and species out of dominant and sub dominant bacterial populations, have been quantified by qPCR which is now routinely used to quantify the bacterial composition of the microbiota of healthy or diseased people or animals [9], [18]. The density of “all-bacteria” in stool samples did not reflect the colonoscopy findings, although one (i.e.; *Bacteroides*) out of the seven species investigated here was found to be higher in cancer group individuals. All these methods are validated, and routinely used [6]. Although pyrosequencing technique, which should be considered as a semi quantitative tool indicated many other bacterial species change, only main dominant and subdominant species were quantified in the present study by qPCR. Furthermore, molecular analyses include species-related differences in probe permeability, and amplification properties, and because of the relatively small number of probes available for analyzing the many uncharacterized gut species [19], we cannot exclude the possibility that we may have missed other significant differences in microbial density. So, this should not exclude possibility of differences between patient groups in terms of bacteria taxa or species that now call for further investigation on the basis of high-throughput sequencing results for cancerous and control microbiota. The rRNA gene–based sequencing can detect the predominant members of the community, but these approaches may not detect the rare members of a community with divergent target sequences. To overcome the limitations of single gene–based amplicon sequencing by pyrosequencing, whole-genome shotgun sequencing has emerged as an attractive strategy for assessing complex microbial diversity in mixed populations [7]. Nevertheless, this is the largest microbiological investigation to have been reported so far, and the large number of subjects enrolled with known colonoscopy and histopathology characteristics make it very robust and could open the way to new pathophysiologic fields and new screening markers.

The reason for an association between *Bacteroides/Prevotella* group density elevation as assessed by qPCR and malignant colon tumors is not clear. All primers used for qPCR in this study were designed to quantify dominant and sub dominant species as suggested by pyrosequencing approach. Whether such microbiological differences found in the present study are cause or consequence of tumour finding at colonoscopy concerns mechanistic approach which was not designed to be analyzed here and requires prospective studies. However, we could speculate that *Bacteroides/Prevotella* group density is probably not the consequence of tumour occurrence because their levels were not correlated with tumour size or disease staging and *Bacteroides* genus species could be detected from washed mucosa suggesting it belongs to mucosal adherent bacteria groups. Primers we designed targeted *Bacteroides* and *Prevotella* genus populations. Changes in *Prevotella* have been only reported in the oral and gastric cavities [20] without any link with tumor growth. In contrast, *Bacteroides* genus populations and more specifically those of *Bacteroides fragilis*, have recently been shown to produce a metalloprotease in colon cancer patients, but not in controls [12] suggesting this species sub population might favor carcinogenesis. It is noteworthy that among the many mechanisms that may mediate associations between microbiota and human health [21]–[22], pro-inflammatory and immune cell activation in colon mucosa are of great importance in relation to malignancy. Some members of the gut microbiota may steer host T-cell responses [13], [15] others may maintain homeostasis of effector helper T-cell populations in the gut [14]. *B. fragilis* has been shown to induce mucosal regulatory T-cell responses in the intestine involving TH17 cell recruitment in experimental models [13]–[14]. Of interest mucosa-adherent *Bacteroides* species in our study appears higher in colon cancer patients than in normal colonoscopy individuals in a proportion linked with mucosal IL17 immunoreactive cell density. This is consistent with our previous study in human that reported TH17 cells overexpression in more than 80% of sporadic colon cancer micro environment [23]. IL17 immunoreactive cells infiltrate more the homologous normal colon mucosa of colon cancer patients than normal tissues in normal colonoscopy individuals as assessed by immunohistochemistry or mRNA qPCR quantification from the mucosa (data not shown). These might suggest T-cell activation can be associated with mucosal IL17 change due to *Bacteroides* as reported in animal models [13]–[14], [21]–[22]. Briefly, these data argued in favor of a disturbed immune response in colon cancer tissues with IL-17 overproduction exacerbating [24]–[25] the disease likely due to *Bacteroides*.

Additional interesting aspect of the microbiota is its potential value as a marker of colon cancer since majority of patients could be identified from an elevation of the *Bacteroides/Prevotella* population with possibility of a quantitative test a cut-off based on a specificity rate. As compared to the colonoscopy so far the elevations of *Bacteroides* in the stool and/or IL17 immunoreactive cells in the normal mucosa appear to be promising sensitive markers.

## Methods

From September 2004 to September 2006, 648 individuals with an average or higher than average risk of CRC (e.g. with history of cancer in relatives or a personal past history of polyps, or any abdominal or intestinal related symptoms or anemia that required colonoscopy), were included in a sample bank collection study. To be eligible for inclusion, the patients had to have no previous history of colon or rectal surgery, of diseases such as cancer, or of inflammatory or infectious injuries of the intestine, and not to need an emergency colonoscopy. Two weeks prior to the colonoscopy, patients were included after giving informed consent and were asked to give a fresh stool sample within 2 weeks up to three days prior to the colonoscopy. The study was approved by ethical committee of Val de Marne Paris-EST area that authorized enrolling patients in all associated centers. All patients received information about the study, its aims, and samples they should give. All information was given by a typed letter written in French and formal consent has been obtained in a triplicate copy form; one of these was conserved by the patient, we keep one copy at the department of clinical research (CIC) and the last copy is conserved by the promoter (National institute of scientific research in medicine-INSERM). So, a formal consent is available for each patient. In all cases stool samples were collected prior to bowel cleansing for colonoscopy. Any particular diets (diabetics, vegetarians) and medications (anti-diabetic drugs, hypocholesterolemics, and laxatives) during this period were recorded. An anesthetist visited the participants at least three days prior to the scheduled colonoscopy. The study period continued until colonoscopy and pathology data had been checked, and the final status could be assigned as “normal colonoscopy”, “Tumour at colonoscopy” or “other abnormalities at colonoscopy”. They were held in a collection bio-bank for pathophysiology or test screening studies, including the one reported here. After gender matching between individuals with tumoral and normal colonoscopies, samples from 180 patients (one cancer patient for 2 individual with normal colonoscopy) who were checked not taking antibiotics with either “normal” or “cancer” findings were subjected to bacteria DNA analysis in the current series.

### Fecal samples and bacterial DNA extraction

Whole fresh stools were collected in sterile boxes, and within 4 hours 10 gr were frozen at −20°C, for analysis. Bacterial DNA was extracted from aliquots of feces, and after the final precipitation, DNA was resuspended in 150 µL of TE buffer, and stored at −20°C for further analysis, as previously described [26].

### **Pyrosequencing analysis from stools**

Bacterial DNA samples from 6 individuals (3 males and 3 females being randomly selected) with a normal colonoscopy, and 6 age- and gender-matched patients with invasive CCR of stage I or II of TNM classification, were used to construct 12 DNA libraries. The following universal 16SrRNA primers were used for the PCR reaction: V3F (TACGGRAGGCAGCAG) [27] and V4R (GGACTACCAGGGTATCTAAT) [28] to target the V3-V4 region, which gives the lowest error rates[29].

Barcode sequences (GsFLX key) TCAG and MIDGsFLX (12 nucleotides) were attached between the 454 GsFLX adaptator sequence and the forward primer V3F. The GsFLX key and the 454 GsFLX adaptator were attached to the reverse primer. The concentration and quality of the PCR products were assessed with Picogreen in order to obtain equal amounts of each of the samples (10^8^ molecules/µl), and then 16S rRNA gene amplicons were sequenced on a Roche GS FLX 454 sequencer (Genoscreen, Lille, France) and processed with standard protocol from manufacturer (http://genoscreen.fr/). To validate the presence of specific bacterial taxa in the 2 groups of patients despite the variability due to the technical process, each DNA sample was sequenced in duplicate. Thus 12 stool bacterial DNA extracts were submitted to pyrosequencing analysis, with two replicates of each. These 24 sets of sequences were submitted to intra individual and inter individual analyses and classical diversity indexes were computed. Common sequences in two duplicates were considered for each individual; then inter individual and inter group analyses were performed according to “Normal” versus “Cancer” status.

### Quantitative PCR analysis

We used a real-time qPCR technique to investigate the difference in bacterial densities within the microbiota between normal and cancer patients' stool (N = 179) and mucosal DNA (N = 44). The primers and probes used in this study have been described elsewhere [26], and are presented in Table S1 supplementary File S1. Real-time qPCR was performed using an ABI 7000 Sequence Detection System with software version 1.2.3 (Applied-Biosystems, Foster City, Ca, USA), and total numbers of bacteria were inferred from averaged standard curves and expressed as log10 value, as previously described [26]. Values of qPCR were obtained per patient and for each component of gut microbiota (total n = 180 patients, 60 with cancer and 119 with normal colonoscopy, one missing sample). To overcome the fact that faecal samples might contain more or less water, the data for each faecal sample was normalized as previously described [26]. The level found for each particular dominant and sub-dominant bacterial population was subtracted from the all-bacteria content, and the results are expressed as the log of the number of bacteria per gram of stool. These assays were used to compare the composition of the intestinal microbiota of the 179 individuals and results are expressed as means ± SD in the normal, and cancer patient groups. In addition, representative colon (N = 32) or rectal (N = 12) normal tissue samples from 22 individuals with normal colon and 22 patients with colon (N = 16) or rectal (N = 6) cancer were submitted to DNA extraction and qPCR quantification according the same procedure to analyze mucosal adherent bacteria component. Colonic or rectal tissues were obtained after surgery; pieces were washed and representative samples were conserved either in formalin for histochemistry or frozen until DNA extraction for human albumin and *Bacteroides* PCR process.

### Immunohistochemistry

Tissue samples were selected for each case (normal and cancer individuals), and paraffin-embedded 4-µm sections were used and immunostaining was performed according to methods described elsewhere [23], [30]. Briefly, the goat anti-human IL-17 antibody (diluted 1∶40) was added for 2 h, and then staining was undertaken using (Vectastain AP kit from Vector Laboratories, Burlingame, CA, USA), and revealed by Naphtol/Fast Red (Sigma-Aldrich). For quantifying the immunostained cells, five well oriented slide samples/individual from normal tissues in either control or colon cancer patients were examined at a magnification of 400× (for a 3-mm-long epithelium sample in each case). Labelled cells per millimeter were determined using an ocular grid. Immunostained cells were counted on 10 consecutive fields and semi-quantitatively scored (as +/-, +, ++ or +++) and classified. For IL17/CD3 double staining, the goat anti-human IL-17 antibody (diluted 1∶40) was added for 2 h, and then staining was undertaken using Vectastain AP kit from Vector Laboratories (Burlingame, CA, USA), and revealed by Naphtol/Fast Red (Sigma-Aldrich). Rabbit anti-human CD3 antibody (diluted 1∶50 in PBS, Dako, France) was added for 1 h, and staining carried out using the ImmPRESS system (Vector Laboratories, Burlingame, CA, USA), and visualization done with DAB substrate. Accordingly, these IL17 and CD3 immunoreactive cells were recognized on red colour and brown colour, respectively. Double stained cells were similarly counted on 10 consecutive fields and semi-quantitatively scored.

### Statistical analyses

- Sequence analysis and phylogenic classification

Raw sequencing reads were quality trimmed according to published recommendations [31]. The trimmed sequences were assigned to taxa using the default settings of the Classifier software [32] to obtain a rapid classification of the bacterial genus. Rarefaction curves and diversity indices were computed for each sample using Vegan package (Community Ecology Package; R package version 1.17-3). The abundance of each taxon was subjected to Principal Component Analysis, with the health status as an instrumental Variable (PCAIV). The link between health status and taxon abundance was reached by a Monte Carlo test with 999 replicates using package ADE4 as described [33]. To avoid the background noise generated by the genus present at lower levels, another PCA was carried out with the best discriminating taxa having more than 1% of observed reads. To assess the impact of colon cancer on the phylogenetic core species as described previously by Tap and colleagues [16]. Blast software was used to assign reads to the representative sequences of Operational Taxonomic Units shared by at least 50% of individual. The impact of cancer on the phylogenetic core was also determined by a Monte Carlo test with 999 replicates.

- qPCR data analysis of individuals' stool samples

All comparisons were performed by means of non parametric tests with a p value of 0.05 as significant. Differences in bacteria levels were searched for between the groups: normal patients and cancers using Mann-Whitney no parametric test.

## Supporting Information

File S1 This file contains description of the whole cohort (Table S1) from which subgroup of patients for microbiota analysis has been selected. The subgroup's characteristics are similar to those of the whole cohort. Additional information on pyrosequencing analyses (Tables S2 and Table S3), primers selected for qPCR of dominant and sub dominant bacteria families (Table S4) as well as correlation with patients' characteristics such as BMI, diet regimen (Figure S1) are given. Additional illustration of bacterial species abundance belonging to the phylogenetic core differentiates cancer patients and healthy individuals (Figure S2) and rarefaction analysis of the pyrosequencing reveals validity of the results (Figure S3). For mucosa-adherent bacteria analysis, characterization of probes for targeting the *Bacteroides* genus in mucosa samples are indicated and sequences of amplification products from mucosa are indicated (Figure S4); *Bacteroides* 16S rRNA and human Albumin genes assessed by Gel electrophoresis are shown on normal and tumoral mucosa (Figure 5).
(DOC)
