# Supplementary material for: Microbial Dysbiosis in Colorectal Cancer (CRC) Patients
Source: PLoS One. 2011 Jan 27;6(1):e16393. doi: 10.1371/journal.pone.0016393 (PMC3029306; doi:10.1371/journal.pone.0016393)
Supplement: File S1 — This file contains description of the whole cohort (Table S1) from which subgroup of patients for microbiota analysis has been selected. The subgroup's characteristics are similar to those of the whole cohort. Additional information on pyrosequencing analyses (Tables S2 and Table S3), primers selected for qPCR of dominant and sub dominant bacteria families (Table S4) as well as correlation with patients' characteristics such as BMI, diet regimen (Figure S1) are given. Additional illustration of bacterial species abundance belonging to the phylogenetic core differentiates cancer patients and healthy individuals (Figure S2) and rarefaction analysis of the pyrosequencing reveals validity of the results (Figure S3). For mucosa-adherent bacteria analysis, characterization of probes for targeting the Bacteroides genus in mucosa samples are indicated and sequences of amplification products from mucosa are indicated (Figure S4); Bacteroides 16S rRNA and human Albumin genes assessed by Gel electrophoresis are shown on normal and tumoral mucosa (Figure 5). (DOC) [file pone.0016393.s001.doc]

**Supplementary file S1**

**Microbial dysbiosis in colorectal cancer (CRC) patients**

*Iradj Sobhani, Julien Tap, Françoise Roudot-Thoraval, Jean-Pierre Roperch; Sophie Letulle; Philippe Langella, Gérard Corthier, Jeanne Tran Van Nhieu and Jean-Pierre Furet*

**Table S1: Characteristics of the whole cohort (N=577** individuals) in the bio-bank

|  | Normal^1^ | Large adenoma^2^ | Cancer^3^ | p (lin) |
| --- | --- | --- | --- | --- |
| Colonoscopy + Pathology (N=) | 426 | 84 | 67 |  |
| Age : m (ds) | 57.1 (11.7) | 62.2 (9.1) | 67.1 (11.6) | 0.001 |
| Sexe M : n (%) | 193 (45.3) | 63 (75.0) | 37 (55.2) | 0.001 |
| Past history polyp : n (%) | 119 (27.9) | 32 (38.1) | 4 (6.0) | 0.011 |
|  |  |  |  |  |
| Diabetes : n (%) | 50 (11.7) | 17 (20.2) | 10 (14.9) | 0.16 |
| Hypercholesterolemia n (%) | 105 (24.7) | 28 (33.3) | 23 (34.3) | 0.044 |
| Familial history of polyps : n (%) | 79 (18.5) | 10 (11.9) | 7 (10.9) | 0.043 |
| Nutrient regimen : n (%) | 66 (15.5) | 19 (22.4) | 13 (19.7) | 0.19 |
| Treatment : n (%) | 315 (73.9) | 71 (84.5) | 51 (76.1) | 0.26 |
| **Reasons for referring to Colonoscopy : n (%)** |  |  |  |  |
| Screening | 180 (42.3) | 29 (34.5) | 13 (19.4) |  |
| Control after polypecyomy | 80 (18.8) | 24 (28.6) | 5 (7.5) | <0.001 |
| Symptoms | 130 (30.5) | 24 (28.6) | 38 (56.7) |  |

^1^- Includes small adenomas<7mm ; ^2^-includes large adenomas only; ^3^- includes invasive cancers, advanced adenomas and large villous tumours

Subgroup of individuals (N and Cancer) chosen for microbiota analysis is representative of the cohort subclasses

**Table S2. 16S rRNA gene sequences processing as a function of samples.**

| Sample | Colonoscopy | Reads | Assignations | Number of detected genera | Gut microbiota core species detected | Core microbiota reads proportion in samples |
| --- | --- | --- | --- | --- | --- | --- |
| 268 mid1 | Cancerous | 51989 | 44803 | 282 | 53 | 27.82% |
| 268 mid2 | Cancerous | 30518 | 26214 | 249 | 53 | 29.82% |
| 414 mid1 | Cancerous | 47789 | 37175 | 348 | 53 | 33.71% |
| 414 mid2 | Cancerous | 47062 | 36844 | 352 | 53 | 31.19% |
| 551 mid1 | Cancerous | 45894 | 37876 | 288 | 54 | 29.06% |
| 551 mid2 | Cancerous | 46427 | 39313 | 279 | 55 | 25.82% |
| 552 mid1 | Cancerous | 51800 | 42228 | 289 | 54 | 31.03% |
| 552 mid2 | Cancerous | 45535 | 37911 | 284 | 54 | 28.92% |
| 722 mid1 | Cancerous | 59517 | 43032 | 346 | 54 | 39.52% |
| 722 mid2 | Cancerous | 44334 | 31912 | 294 | 52 | 38.67% |
| 825 mid1 | Cancerous | 54104 | 44942 | 319 | 49 | 18.95% |
| 825 mid2 | Cancerous | 43283 | 36005 | 281 | 49 | 20.18% |
| 192 mid1 | Normal | 37064 | 28507 | 314 | 54 | 42.96% |
| 192 mid2 | Normal | 50184 | 39301 | 312 | 55 | 40.69% |
| 500 mid1 | Normal | 35007 | 26491 | 300 | 55 | 38.02% |
| 500 mid2 | Normal | 43574 | 32587 | 314 | 55 | 35.84% |
| 510 mid1 | Normal | 39752 | 34768 | 287 | 55 | 23.89% |
| 510 mid2 | Normal | 39199 | 34849 | 279 | 55 | 19.40% |
| 542 mid1 | Normal | 184454 | 161576 | 433 | 52 | 20.99% |
| 542 mid2 | Normal | 49951 | 44441 | 293 | 51 | 17.18% |
| 568 mid1 | Normal | 42771 | 27195 | 298 | 49 | 56.82% |
| 568 mid2 | Normal | 34546 | 21535 | 232 | 50 | 53.37% |
| 820 mid1 | Normal | 45196 | 36299 | 293 | 48 | 29.38% |
| 820 mid2 | Normal | 40831 | 32906 | 265 | 51 | 29.64% |

A total of 1210781 reads were obtained for all samples from pyrosequencing and 978710 of them were assigned by classifier software. A total number of 819 genera were distributed among all samples with few variations between replicates. Fifty six species hat belonged to the phylogenetic core of microbiota were detected in the datasets and they represented the third of reads which was consistent with of the phylogenetic core as previously described [16].

**Table S3:** **Diversity of the gut microbial community between the normal (N) and cancer (Ca) individuals.**

| *Sample_ID* | *health_status* | *Common_replicates_genus* | *Simpson* | *Shannon* |
| --- | --- | --- | --- | --- |
| 192 | N | 235 | 0.924 | 3.398 |
| 268 | Ca | 180 | 0.908 | 2.985 |
| 414 | Ca | 252 | 0.945 | 3.565 |
| 500 | N | 225 | 0.917 | 3.218 |
| 510 | N | 202 | 0.89 | 3.048 |
| 542 | N | 262 | 0.914 | 3.041 |
| 551 | Ca | 204 | 0.948 | 3.518 |
| 552 | Ca | 208 | 0.947 | 3.459 |
| 568 | N | 178 | 0.944 | 3.434 |
| 722 | Ca | 213 | 0.959 | 3.706 |
| 820 | N | 192 | 0.77 | 2.48 |
| 825 | Ca | 207 | 0.933 | 3.329 |

Number of common replicates genus, Simpson and Shannon diversity indexes are indicated.

**Table S4**: **Group and species-specific 16S rRNA gene-targeted primers and probes.**

| ***Target organism*** | ***Primer***  ***and probe*** | | ***Sequence 5’- 3’*** | |  |
| --- | --- | --- | --- | --- | --- |
| *All-bacteria* **(*)** | | F_Bact 1369 | | CGG TGA ATA CGT TCC CGG | |
|  | | R_Prok1492 | | TAC GG**C** TAC CTT GTT ACG ACT T | |
|  | | **P_TM1389F** | | **6FAM-CTT GTA CAC ACC GCC CGT C** | |
| *C. leptum* | | F_Clept 09 | | CCT TCC GTG CCG SAG TTA | |
|  | | R_Clept 08 | | GAA TTA AAC CAC ATA CTC CAC TGC TT | |
|  | | **P-Clep 01** | | **6FAM-CAC AAT AAG TAA TCC ACC** | |
| *Bifidobacterium* | | F_Bifid 09c | | CGG GTG AGT AAT GCG TGA CC | |
|  | | R_Bifid 06 | | TGA TAG GAC GCG ACC CCA | |
|  | | **P_Bifid** | | **6FAM-CTC CTG GAA ACG GGT G** | |
| *C. coccoides* | | F_Ccoc 07 | | GAC GCC GCG TGA AGG A | |
|  | | R_Ccoc 14 | | AGC CCC AGC CTT TCA CAT C | |
|  | | **P_Erec482(*)** | | **VIC-CGG TAC CTG ACT AAG AAG** | |
| *Bacteroides/* | | F_Bacter 11 | | CCT WCG ATG GAT AGG GGT T | |
| *Prevotella* | | R_Bacter 08 | | CAC GCT ACT TGG CTG GTT CAG | |
|  | | **P_Bac303(*)** | | **VIC-AAG GTC CCC CAC ATT G** | |
| *E. coli* | | E.coli F | | CAT GCC GCG TGT ATG AAG AA | |
|  | | E.coli R | | CGG GTA ACG TCA ATG AGC AAA | |
| *Lactobacillus/* | | F_Lacto 05 | | AGC AGT AGG GAA TCT TCC A | |
| *Leuconostoc/ Pediococcus* | | R_Lacto 04 | | CGC CAC TGG TGT TCY TCC ATA TA | |
| *F. prausnitzii* | | Fprau 07 | | CCA TGA ATT GCC TTC AAA ACT GTT | |
|  | | Fprau 02 | | GAG CCT CAG CGT CAG TTG GT | |

(*) modified from reference

Primers and probes were constructed to target all dominant and subdominant bacterial species in accordance to the pyrosequencing results. Methods have been previously described [20, 22]. Probe sequences are in bold.

**Figure S1**. **Effects of age, BMI, medications, diet and cancer staging on *Bacteroides*  elevation in faecal samples.**

**Log (*Bacteroides* – All Bacteria) as obtained by qPCR technique from faecal DNA**

0

- 5

- 4

- 3

- 2

- 1

A

D

**Every day alimentary diet**

A

Ac

Ad

La

Ve

**Medication**

**medication**

10

20

30

40

50

60

70

80

90

**Age (Yr)**

**Y = 1.565 - .004 * X; R^2 = .005P=0.46**

0

- 1

- 2

- 3

- 4

- 5

**A**

0

- 1

**Stage**

**in TNM classification**

**I-II III IV**

**B**

**C**

**D: Diabetes; Ve.: Vegetarian; Ac: antagonist of cholesterol; La: Laxatives; Ad: anti diabetes ; A: any;**

**Figure S2: Bacterial species abundance belonged to the phylogenetic core differentiates cancer patients and healthy individuals.**

d = 2

Cancer

Normal

Principal Component Analysis, based on the 16S rRNA gene sequence abundance of 10 discriminates phylogenetic core species, was carried out with 6 normal individuals (red points) and 6 cancer patients (black points) with two replicates. Two first components (PC1 and PC2) were plotted and represented 57.95 % of whole inertia. Individuals were clustered and centre of gravity computed for each class. Following core species bacteria decrease in cancer patient:

*Bifidobacterium longum*; BG; AY675246

*Clostridium clostridioforme*; 1-53; AY169422

*Ruminococcus sp. DJF_VR66*; EU728790

*Ruminococcus bromii*; L2-63; EU266549

**Figure S3.** **A rarefaction analysis of the pyrosequencing data.**

Common sequences from duplicates are included in the analyses comparing normal individuals (black) and cancer (red) patients. The plateau is obtained from 40000 sequences per individual.

**Figure S4. Characterization of probes for targeting the *Bacteroides* genus in mucosa samples.**

Sequences of product after Forward Primer amplification:

5’ – ACGGTCCAAACTCCTACGGGAGGCAGCAGTGAGGAATATTGG

TCAATGGACGAGAGTCTGAACCAGCCAAGTAGCG – 3’


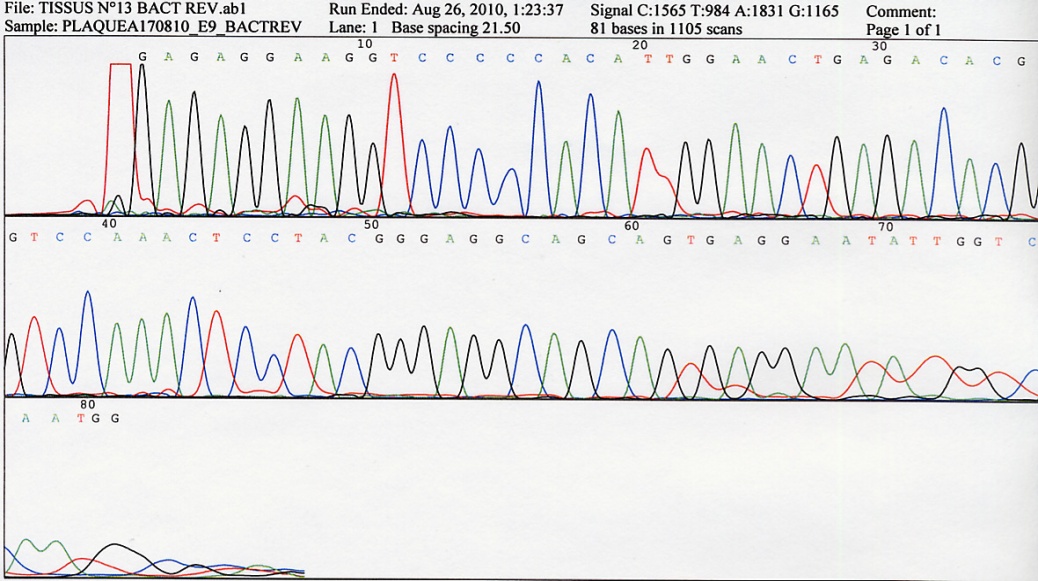

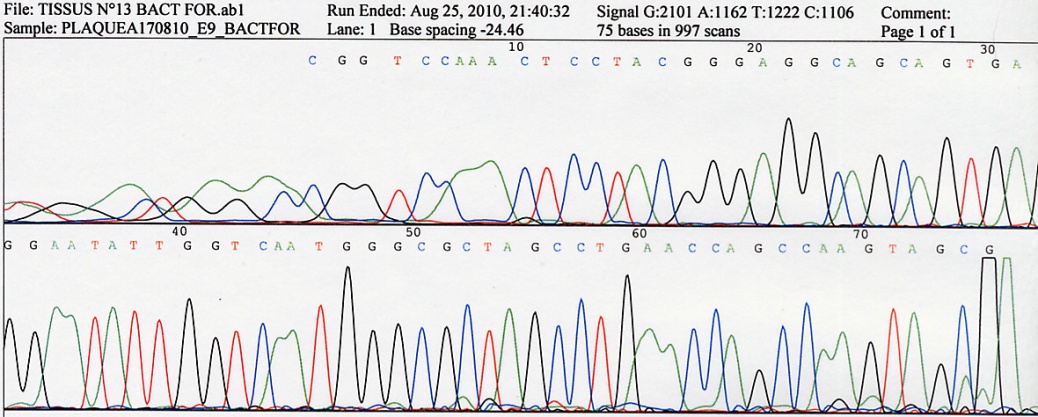


Sequences of Product after Reverse Primer amplification:

5’-GAGAGGAAGGTCCCCCACATTGGAACTGAGACACGGTCCA

AACTCCTACGGGAGGCAGCAGTGAGGAATATTGG – 3’

**Bacteroides**

**Forward primer**

5’-GAGAGGAAGGTCCCCCAC-3’

T°m=60°C 18 bases

**Reverse Primer**

5’-CGCTACTTGGCTGGTTCAG-3’

T°m=60°C 19 bases

**Amplification Product of 108 bases: 100% homology with *Bacteroides***

5’-GAGAGGAAGGTCCCCCACATTGGAACTGAGACACGGTCCAAACTCCTACGGGAGGCAGCAGTGA

GGAATATTGGT CAATGGACGAGAGTCTGAACCAGCCAAGTAGCG-3’

DNA has been extracted and amplification was performed as previously described. For PCR Sybr Green technique was used on 7900 HT – Applied biosystems : 400 nmol Primers(For/Rev) were used after Hot start for 15 min at +95°C, amplification lasted on 48 cycles, followed by 15 sec at +95°C, and 1 min at +60°C; Setup was done using Sybr Green detector with specific marker for albumin and Bacteria and Real time dissociation curve construction. Following primers were used:

Albumin (77 pb amplicon),

forward 5’-GGGATGGAAAGAATCCTATGCC-3’

reverse: 5’-GGACAGGCTGACCCCAAATTCT-3’

*Bacteroides* (108 pb amplicon):

forward, 5’-GAGAGGAAGGTCCCCCAC-3’

reverse: 5’-CGCTACTTGGCTGGTTCAG-3’

All PCR products have been submitted to the whole sequencing process. Albumin and *Bacteroides* PCR products were obtained in either tissue samples from normal mucosa in controls, and in normal and tumoral tissues in colon cancer patients (see Figure 2 in the manuscript). These PCR products were isolated from stool DNA samples. Interestingly, *Bacteroides* and albumin genes’ products appeared from 23-25 cycles amplification with *Bacteroides* being more expressed in cancer patients’ tissues than in normal individuals as assessed on gel analysis (see also Figure 2).

While *Bacteroides* gene amplification from stools appeared from 13-15 cycles and albumin from stools close to 30-35 cycles. These suggested *Bacteroides* were highly present in stools as compared to the human albumin PCR product in stools, it can be considered as adherent to the mucosa in very lower proportion as assessed by PCR products. Specific tissue adherent *Bacteroides* appears more in tumour tissues than in normal.

**Figure S5. Gel electrophoresis of *Bacteroides* 16S rRNA and human Albumin genes.**

12 13 14 15 16 17 18 19

***Bacteroides*: 108 pb**

**Albumin: 77 pb**


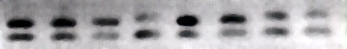


Note that *Bacteroides* amplification products in normal tissue from normal colonoscopy individuals (line 18 and 19) are lower than in tissues (lines 12, 14, 16 tumoral tissues and 13, 15, 17 homologous normal tissues) in colon cancer patients.
